# HIV-AIDS stigma and discrimination in health care sector in Belarus

**DOI:** 10.1186/1742-4690-9-S1-P81

**Published:** 2012-05-25

**Authors:** Vera Ilyenkova, Svetlana Kunitskaya, Irina Eramova

**Affiliations:** 1 World Health Organisation, Regional Office for Europe, Communicable diseases, Minsk, Belarus; 2 Belarusian State Medical University, Public Health, Minsk, Belarus; 3 World Health Organisation, Regional Office for Europe, Communicable diseases, Copenhagen, Denmark

## Background

Stigma and discrimination are barriers to effective HIV treatment and care in Belarus. Results of the Stigma Index Survey conducted among people living with HIV (PLHIV) reveal that 40.5% of respondents experienced disclosure of their diagnosis and confidentiality breach by health care workers (HCW); 15.5% were refused medical care.

## Methods

Public health department of Belarusian State Medical University and NGO “Fialta” investigated the extent and possible reasons of HIV-related stigma in health sector, by conducting a survey on knowledge, misconceptions, attitudes and motivations regarding HIV/AIDS among 40 HCW not routinely involved in HIV-care. This was followed by two one-day sensitizing workshops addressing Belarusian law on HIV/AIDS, emphasizing medical rules/regulations, patients’ rights and confidentiality, stigma and discrimination, including sensitizing role plays.

## Results

Though the sample size was small, research findings indicate needs to address levels of stigma and discrimination among HCW in Belarus: a quarter of respondents are not willing to provide services to PLHIV at all; more than half are inclined to violate patients’ rights and test patients for HIV without informed consent; majority revealed some negative stereotypes in regards to PLHIV that lead to discrimination outside their professional duties (for example, changing HIV-positive hairdresser, advising their children to reduce contacts with HIV-positive schoolmate); about 2/3 would isolate PLHIV if they are not their friends or relatives. Possible reasons from workshops’ findings: HCW don’t realize what stigma is and don’t know their actions/attitudes are discriminative; lack of knowledge about HIV-infection resulted in fear of contact with PLHIV; negative stereotypes regarding PLHIV exist among population as they “traditionally” represent risk-groups (injection drug users, commercial sex workers, etc.), thus, “they are immoral and dangerous”.

## Conclusions

Introducing stigma-reducing interventions (sensitizing workshops, educational briefings) in health sector would help to improve the situation. Conducting in-depth interviews among HCW is necessary to analyse stigma-related issues more thoroughly.

